# Vitamin D_3_ supplementation using an oral spray solution resolves deficiency but has no effect on VO_2_ max in Gaelic footballers: results from a randomised, double-blind, placebo-controlled trial

**DOI:** 10.1007/s00394-016-1202-4

**Published:** 2016-03-25

**Authors:** Joshua J. Todd, Emeir M. McSorley, L. Kirsty Pourshahidi, Sharon M. Madigan, Eamon Laird, Martin Healy, Pamela J. Magee

**Affiliations:** 1 Northern Ireland Centre for Food and Health, University of Ulster, Coleraine, UK; 2 Irish Institute of Sport, Sports Campus Ireland, Abbotstown, Dublin 15, UK; 3 Institute of Molecular Medicine, Trinity College, Dublin, UK; 4 Department of Medicine, Trinity Centre for Health Science St. James’s Hospital, Dublin, UK

**Keywords:** Vitamin D, VO_2_ max, Athletes, Randomised controlled trial, Football

## Abstract

**Purpose:**

Vitamin D inadequacy is a global health concern in athletes as well as the general population. Whilst the role of vitamin D in skeletal health is well defined, there remains uncertainty over whether vitamin D supplementation has an added benefit beyond bone health.

**Methods:**

This randomised placebo-controlled trial in healthy male and female Gaelic footballers (*n* = 42) investigated the effect of vitamin D_3_ supplementation [3000 IU (75 µg) daily for 12 weeks, via an oral spray solution] on VO_2_ max which was the primary outcome measure. Secondary outcomes included skeletal muscle and lung function.

**Results:**

Supplementation significantly increased total 25-hydroxyvitamin D concentrations compared to the placebo group (mean ± SD change from baseline, 36.31 ± 32.34 vs. 6.11 ± 23.93 nmol/L, respectively; *P* = 0.006). At baseline, 50 and 22 % of footballers presented with vitamin D insufficiency (31–49 nmol/L) and deficiency (<30 nmol/L), respectively. Total 25-hydroxyvitamin D concentration did not significantly correlate with any measure of physical performance. Analysis of covariance (ANCOVA) models demonstrated that vitamin D supplementation over 12 weeks had no significant effect on VO_2_ max (*P* = 0.375), vertical jump height (*P* = 0.797), left and right handgrip strength (*P* = 0.146 and *P* = 0.266, respectively), forced vital capacity (*P* = 0.573) or forced expiratory volume at 1 s (*P* = 0.665), after adjusting for confounders. The high prevalence of vitamin D inadequacy observed in this cohort of collegiate Gaelic footballers supports the need for vitamin D supplementation during wintertime to avoid being at risk of poor bone health.

**Conclusions:**

Twelve-week daily supplementation with 3000 IU (75 µg) vitamin D_3_ successfully resolved deficiency but did not have any significant effect on VO_2_ max, skeletal muscle or lung function.

## Introduction

Vitamin D insufficiency and deficiency can be defined as a total serum 25-hydroxyvitamin D (25[OH]D) concentration below 50 and 30 nmol/L, respectively [1]. Such low total 25(OH)D concentrations are widespread, and a growing number of studies around the globe have identified this health concern in athletes [2–5]. Vitamin D and its metabolites are renowned for their pivotal role in establishing musculoskeletal health during childhood and adolescence and in hindering the development of skeletal pathology [6]. In addition, a growing body of evidence has demonstrated the importance of vitamin D beyond bone health on immune, cardiopulmonary and skeletal muscle function [7].

Biologically inactive total 25(OH)D is comprised of 25(OH)D_2_ and 25(OH)D_3_, compounds that are both formed following hepatic hydroxylation of vitamin D_2_ (ergocalciferol) and vitamin D_3_ (cholecalciferol) [8]. Ergocalciferol is derived from fungi exposed to ultraviolet B radiation (UVB) [9]. In humans, cholecalciferol is produced following exposure of cutaneous cells to UVB at a wavelength of 290–315 nm and is also present in oily fish, eggs and liver [10]. Calcitriol (1,25-dihydroxyvitamin D (1,25[OH]_2_D) is formed subsequent to renal hydroxylation of 25(OH)D, and it is this hormonally active compound that has been implicated in numerous processes involving the immune, cardiovascular and respiratory systems [11]. Binding of 1,25[OH]_2_D to its nuclear receptor (VDR) results in the formation of a VDR-retinoid X receptor (RXR) heterodimer that enables 1,25(OH)_2_D to act as a transcriptional regulator through binding to response elements located in the DNA of vitamin D target tissues [12].

In observational studies of both athletes and non-athletes, total 25(OH)D concentration has been positively associated with measures of aerobic fitness although findings have not been consistent [13–19]. One such measure is VO_2_ max, the maximal volume of oxygen utilised, per minute, during exhaustive exercise, which is considered the gold-standard measure of aerobic fitness and is related to distance covered in field games [20]. VO_2_ max is determined by a range of overriding factors including cardiac output, oxygen transit time and oxygen saturation [21].

A major limitation of existing observational research in this area is its inability to determine causality, and therefore, it is not known whether total 25(OH)D concentration is a cause of increased VO_2_ max or simply a result of reverse causation, perhaps owing to increased outdoor training time [22]. Despite the paucity of randomised controlled trials investigating this relationship, there is significant evidence of mechanisms by which vitamin D may influence factors that determine VO_2_ max [23–25]. One such mechanism is the ability of 1,25(OH)_2_D to suppress mRNA expression of hepcidin, the negative regulator of systemic iron concentration, an action which has been associated with increased expression of ferroportin in hepatocytes and monocytes [24]. Ferroportin is the sole iron export protein in humans and plays a crucial role in maintaining erythropoiesis, a contributory factor to VO_2_ max [26–28]. Studies in both healthy adults and patients with chronic kidney disease have demonstrated that vitamin D supplementation significantly decreases systemic hepcidin concentrations [24, 25]. Collectively, these findings raise the question as to whether optimising total 25(OH)D concentrations has a beneficial effect on VO_2_ max in athletes.

The primary aim of the current study was to test whether athletes who received daily vitamin D_3_ supplementation at 3000 IU (75 µg) demonstrated a significant increase in VO_2_ max compared to those provided with a placebo. Secondary aims were to determine whether supplementation influenced skeletal muscle and lung function.

## Experimental methods

This 12-week parallel group, double-blind, randomised, placebo-controlled trial was conducted at the University of Ulster, Coleraine, at a latitude of 55°N between the months of November 2014 and April 2015. All procedures were approved by the University’s Research Ethics Committee (REC/14/0087), and the study was registered at www.clinicaltrials.gov (NCT02278172) and conducted in accordance with the Declaration of Helsinki. The protocol consisted of a series of appointments to obtain fasted blood samples and other physical measurements before and after a 12-week intervention. Nutritional and exercise intervention studies in athletes and healthy adults have demonstrated that 12 weeks is a suitable duration to observe a meaningful change over time in VO_2_ max [29–31]. Yet the dose and duration of vitamin D supplementation required to have a significant impact on VO_2_ max have not been investigated to date.

### Subjects

Apparently healthy male and female athletes over the age of 18 were considered suitable for inclusion. Exclusion criteria were as follows: not a member of a university sports team; vitamin D supplementation and/or iron supplementation in the 30 days prior to baseline measurements; health concern(s)/physical disabilities identified by the screening questionnaire that would prevent successful completion of the study; consumption of medication(s) known to influence vitamin D metabolism; vegan athletes; sun-bed users; those who had been on a sun holiday in the 30 days prior to baseline measurements; those planning a sun holiday for during the time frame of the study. Gaelic footballers from the university team (*n* = 72) completed a screening questionnaire in the first instance. A total of 42 Gaelic footballers (*n* = 18 males and *n* = 24 females) were deemed eligible for inclusion and provided informed consent before commencing the study.

### Supplements and compliance

An independent clinical trials manager used MINIM software [32] to randomise recruited athletes into vitamin D (VD) or placebo (PL) groups, stratified by sex and with an allocation ratio of 1:1. All subjects and researchers were blinded to the allocations until completion of the study and subsequent data analysis. Footballers allocated to the VD group received an oral spray solution providing 3000 IU(75 µg) vitamin D_3_ per spray, whereas those allocated to the PL group received an oral spray solution that did not contain vitamin D but was identical in appearance, smell, taste and from the same brand (BetterYou Ltd, Barnsley, UK). The dose provided was deemed suitable to raise total 25(OH)D concentrations to within the suggested range for extra-skeletal actions of vitamin D (75–100 nmol/L) [33–35]. The vitamin D_3_ content of supplements was verified by an independent laboratory using high-performance liquid chromatography (Eurofins Product Testing, Cheshire, UK). Footballers were instructed to administer a single spray, targeting the buccal membrane, on a daily basis throughout the intervention and to return their used spray bottle at their final appointment. Percentage compliance was determined by applying the following equations.

1 $$D = \left( {{\text{f}} - {\text{b}}} \right) - {\text{e}} \div s.$$

2 $$C = D \div {\text{d}} \times 100.$$

In Eqs. 1 and 2, *D* is number of days the spray was taken; f refers to the filled weight of the spray bottle, and b is the empty spray bottle weight. e is the weight of the spray bottle upon study completion with *s* referring to the weight of each spray. In Eq. 2, C is percentage compliance and d represents the number of days on intervention. Filled weights were based upon the manufacturer’s specifications and the average weight of a random sample of 10 oral spray bottles from the supplied batch.

### Blood collection and processing

Participants were instructed to fast from 10 pm the night prior to blood sampling, and regular water intake was encouraged. Fasted blood samples were obtained from the antecubital fossa using a 21-gauge butterfly needle and 8 mL serum and 9 mL ethylenediaminetetraacetic acid (EDTA) plasma vacutainer tubes (Greiner Bio-One GmbH, Kremsmunster, Austria). Following inversion, serum samples were allowed to clot for <60 min and plasma samples placed in refrigeration until centrifugation. Tubes were centrifuged at 2200 rpm for 15 min at 4 °C to allow separation of whole blood into its respective components. Following separation, serum and plasma samples were pipetted into 0.5 mL aliquots and stored at −80 °C until further analysis.

### Blood analyses

Liquid chromatography-tandem mass spectrometry (LCMS-MS) (API 4000; AB SCIEX) was used to quantify serum 25(OH)D_2_ and 25(OH)D_3_ concentrations, using a commercially available assay (Chromsystems Instruments and Chemicals GmbH; MassChrom 25-OH-Vitamin D_3_/D_2_). This analysis was undertaken at the Biochemistry Department of St James’ Hospital Dublin, a laboratory that complies with the Vitamin D External Quality Assessment Scheme and use of the National Institute of Standards and Technology 972 vitamin D standard reference material. The respective inter- and intra-assay coefficients of variation were 6.5 and 7.5 %. Plasma intact parathyroid hormone concentrations were measured, in duplicate, using a commercially available enzyme-linked immunosorbent assay (MD Biosciences Inc., Minnesota, USA). Intra and inter-assay coefficients of variation were 6.8 and 6.2 %, respectively. Calcium, albumin and creatinine concentrations were also measured in duplicate and assessed using an ILab 650 clinical biochemistry analyser (Instrumentation Laboratory, Massachusetts, USA). Intra-assay coefficients of variation were 0.65, 0.85 and 1.65 %, respectively. Intact parathyroid hormone and adjusted calcium concentrations were measured in order to ensure there were no adverse effects of the intervention such as hypoparathyroidism or hypercalcaemia [36]. Impaired renal function can lead to deleterious effects on vitamin D metabolism, and therefore, the renal function of athletes’ was evaluated by estimating glomerular filtration rate (eGFR) adjusted for fat-free mass [37, 38]. This was quantified from serum creatinine concentrations using the Modification of Diet in Renal Disease (MDRD) equation [39].

### Skeletal muscle function

Average handgrip strength (kg) was measured using a dynamometer assembled at handgrip position 2 as default with shoulder flexion at 0º and elbow and wrist fully extended (Jamar Plus+, Patterson Medical, Warrenville, IL, USA) [40]. Footballers held the device alongside their body and gripped maximally a total of 3 times. Average handgrip strength was calculated for analysis. Vertical jump height (cm) was measured using a calibrated electronic jump mat (FSL Electronics Ltd, Cookstown, UK). Footballers performed a counter-movement jump a total of 3 times, with best recorded jump height used for statistical analysis [41]. A standardised rest period of 10 s was given between repeats of both handgrip and vertical jump tests.

### Lung function

Footballers’ lung function was assessed using a calibrated MicroLab portable spirometer (Carefusion Corporation, San Diego, CA, USA). Forced vital capacity (FVC) and forced expiratory volume at 1 s (FEV_1_) were quantified by exhaling maximally into a 1-way disposable mouthpiece, and a minimum of 3 repeats was performed in order to derive average values.

### Maximal oxygen consumption

Footballers refrained from heavy training/competition for at least 24 h prior to exercise testing, in order to control for last-bout effects. Pre- and post-intervention, subjects completed an incremental exercise test that was designed to elicit a VO_2_ max response within the capabilities of the treadmill (MERC-C, WOODWAY GmbH, Germany). Gas analysis was performed using a metabolic cart that has been shown to give reliable measurements (Metalyzer 3B, CORTEX Biophysik GmbH, Germany) with calibrations for ambient conditions, analyser volume and concentrations of oxygen and carbon dioxide performed on a daily basis, prior to testing [42]. Footballers wore an FT1 heart rate monitor (Polar Electro Ltd, Warwick, UK) and face mask with triple V insert. Following a standardised warm-up at 5 km/h (1 % incline), the test began at a running speed of 8 km/h (1 % incline) and speed increased by 1 km/h every minute until a running speed of 17 km/h was attained. At this point, running speed remained constant; however, incline increased by 1 % each minute until VO_2_ max was achieved. The test was terminated upon volitional exhaustion or if any two of the following criteria were met: respiratory exchange ratio >1.15; oxygen plateau observed (i.e. no increase in oxygen consumption despite an increase in workload); heart rate ±10 bpm of age-predicted maximum (208 − 0.7 × age) [43]. Achievement of VO_2_ max was further verified by determining post-exercise lactate concentrations, using a Lactate Pro device as per the manufacturers’ recommendations (Arkray Inc, Kyoto, Japan). Peak lactate concentrations are reached between 3 and 8 min post-exercise [44]; therefore, at 5 min, following cessation of the VO_2_ max test, a 6-µL capillary blood sample was obtained for lactate analysis. The lactate response to exercise may also vary according to age and sex. To account for this a blood lactate concentration >9 mmol/L in males and >7 mmol/L in females was considered as evidence of significant anaerobic metabolism [45].

### Body composition

Footballers’ height and weight were measured using a stadiometer and calibrated scales. Fat mass (FM) and fat-free mass (FFM) were measured by whole-body densitometry pre- and post-intervention (BOD POD, Life Measurement Inc, Concord, CA). The validity of this method has been reported extensively elsewhere [46]. Footballers were asked to refrain from heavy physical activity for 24 h, and testing was performed following an overnight fast. Body mass was calculated using a calibrated electronic scale. Body volume was determined by air displacement plethysmography, corrected for predicted thoracic gas volume. The plethysmography chamber was calibrated using a 49.385-L cylinder as per manufacturers’ instructions. Percentage fat mass (%FM) was calculated using the Siri equation [47]. Footballers wore tight-fitting clothing (i.e. swimsuit or compression garment with silicone swimming cap), to ensure an accurate measurement of body volume, and were instructed to sit upright and remain still throughout the test. A minimum of two measurements was taken per athlete. FM and FFM (kg) were adjusted for athlete’s height (m^2^) and presented as fat mass index (FMI) and fat-free mass index (FFMI) (kg/m^2^) [48].

### Dietary assessment

In order to estimate dietary vitamin D intake, footballers completed a validated food frequency questionnaire (FFQ) [49]. These data were collected on a single occasion due to the negligible contribution of dietary sources to total 25(OH)D concentration and the low intake of such foods in the Western diet [50, 51], despite a growing range of vitamin D-fortified products. Researchers asked players a series of questions relating to their consumption of food items known to contain vitamin D, with a photographic food atlas used to estimate portion sizes [52].

### Physical activity

The validated Recent Physical Activity Questionnaire (RPAQ), capable of assessing physical activity for the previous 4 weeks, was completed pre- and post-intervention to control for a change in moderate-vigorous physical activity during the study [53]. Participants completed RPAQ questionnaires during appointments, and the researcher present queried any ambiguous responses prior to data entry.

### Statistical analysis

**Fig. 1:**
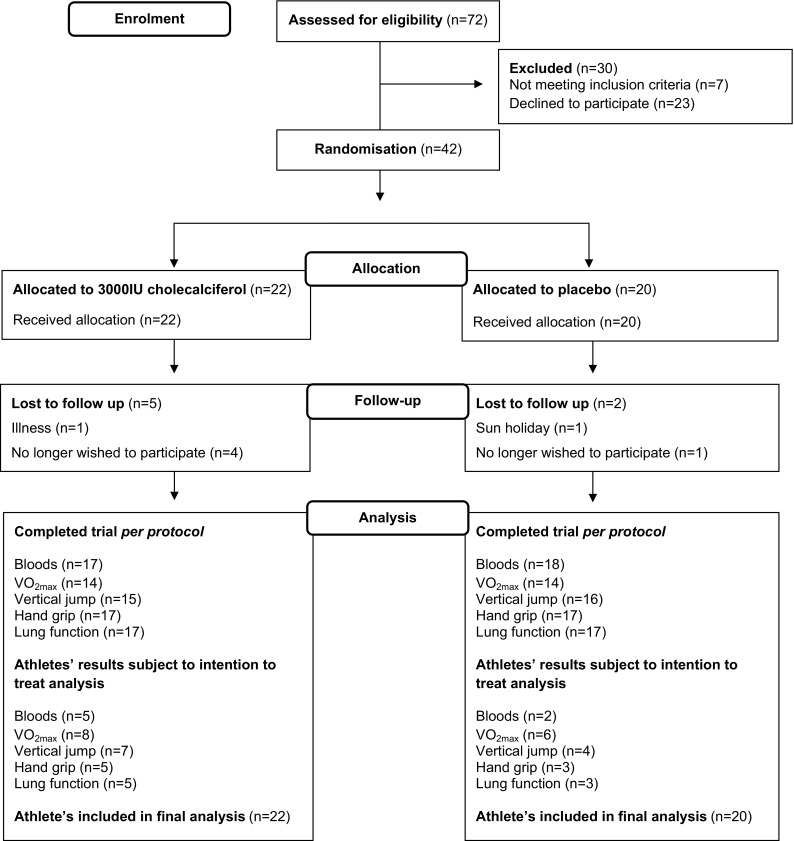
CONSORT flow diagram. A total of 72 Gaelic footballers were assessed for eligibility with 30 excluded due to not meeting the inclusion criteria (*n* = 7) or no longer wishing to participate (*n* = 23). Remaining Gaelic footballers (*n* = 42) were randomised to receive an oral spray solution containing either 3000 IU of vitamin D_3_ (*n* = 22) or placebo (*n* = 20). A total of 7 athletes were lost to follow-up owing to illness (*n* = 1) unrelated to the intervention, sun holiday (*n* = 1) or no longer wishing to participate (*n* = 5). A total of 35 athletes completed the study per protocol (vitamin D treatment group *n* = 18 and placebo *n* = 17). All footballers randomised at baseline were included in the final analysis (*n* = 42)

An a priori power calculation with a two-sided 5 % significance level and power at 95 % determined that 35 athletes were required to observe a statistically significant 3.5 mL/kg/min increase in VO_2_ max (G Power version 3.1) [54]. The final number of recruited athletes (*n* = 42) took into account an estimated dropout rate of 20 %. All statistical analyses were performed using the Statistical Package for the Social Sciences (SPSS) with significance set at *P* < 0.05 throughout (IBM SPSS Statistics for Windows, version 21.0, IBM Corp, Armonk, NY). In accordance with the Consolidated Standards of Reporting Trials (CONSORT) guidelines, analyses were conducted using the intention-to-treat (ITT) principle thereby including all athletes randomised at baseline (*n* = 42) [55]. Missing data for physical and biochemical measures were deemed to be missing completely at random, owing to injury or illness unrelated to the intervention, justifying the use of multiple imputation. The Shapiro–Wilk test was used to determine whether data followed a normal distribution and skewed variables were transformed, using the logarithmic function, to attain a normal distribution prior to multiple imputation and further analysis. Transformations were applied to total 25(OH)D, creatinine and PTH concentrations as well as age and change in moderate-vigorous physical activity and FFMI. Multiple imputations consisted of 40 imputed data sets with pooled data used for subsequent analysis [56]. Imputed data are outlined in Fig. 1. Descriptive statistics were used to present participant characteristics at baseline. An independent *t* test or a Chi-square test was utilised to test for differences between VD and PL groups at baseline. ANCOVA models were used to assess the effect of intervention on VO_2_ max and secondary outcome measures. Prognostic covariates were selected a priori for each model based upon evidence of a significant interaction between the covariate and dependent variable in question [57–60]. Reliability of repeated skeletal muscle function tests was assessed by pooling pre- and post-intervention data in order to determine the standard error of measurement (SEM) and intra-class correlation coefficient (ICC) [61]. These analyses were performed using the reliability test function in SPSS and SEM calculated by applying Cronbach’s α to following equation. ${\text{SEM}} = {\text{SD}}\sqrt {(1 {-} r_{xx}^{2} )}$ where SEM is standard error of measurement, SD refers to the standard deviation of test scores, and *r*_*xx*_ refers to Cronbach’s α as the reliability measure of test scores [62].

## Results

A total of 7 footballers were absent to follow-up (*n* = 5 VD and *n* = 2 PL) due to sun holidays or illness unrelated to the intervention. Use of ITT did not change the outcome of the study when compared with *per protocol* analysis. There was no significant difference in the ratio of male to female subjects between groups (males *n* = 12 and females *n* = 10 VD; males *n* = 6 and females *n* = 14 PL) *P* = 0.857. The participant flow throughout the study is summarised in Fig. 1. Footballers’ biochemical and physical characteristics at baseline and week 12 are provided in Table 1. The average rate of compliance to the intervention was 95 %. At baseline, 50 and 22 % of athletes presented with vitamin D insufficiency (31–49 nmol/L) and deficiency (<30 nmol/L), respectively. The effect of the intervention on outcome measures at week 12, after adjusting for covariates, is detailed in Table 2. Supplementation significantly increased total 25(OH)D concentrations in the VD group compared to PL group, *P* = 0.006, and resolved vitamin D deficiency in all athletes allocated to VD. There was no correlation between total 25(OH)D and measures of physical performance at either time point, *P* > 0.05. Furthermore, supplementation with vitamin D_3_ did not significantly increase VO_2_ max compared to the PL group, *P* = 0.375. Vitamin D_3_ supplementation did not significantly increase vertical jump height when compared to the PL group, *P* = 0.797, and also had no significant effect on left or right handgrip strength, *P* = 0.146 and *P* = 0.266, respectively. SEM and ICC results for vertical jump height (SEM = 1.51, *r* = 0.95), left and right handgrip strength (SEM = 2.06, *r* = 0.96 and SEM = 2.34, *r* = 0.95, respectively) indicated a strong internal consistency across repeated skeletal muscle function tests. Supplementation with vitamin D_3_ did not have a significant effect upon either measure of footballers lung function compared to those allocated to PL, FVC *P* = 0.573 and FEV_1_ *P* = 0.665. There were no adverse health effects to supplementation demonstrated by normal adjusted calcium and PTH concentrations at week 12.

**Table 1:** Footballers’ characteristics at baseline and week 12 expressed as mean ± standard deviation

Treatment group	Vitamin D (*n* = 22)		Placebo (*n* = 20)		*P*	*P*
Measure	Baseline	Week 12	Baseline	Week 12		
Age, year	20 ± 2	–	20 ± 2	–	0.819	–
Height, cm	171.39 ± 8.65	–	165.65 ± 10.18	–	0.048^a^	–
Weight, kg	70.52 ± 11.49	71.1 ± 11.91	61.92 ± 10.69	62.30 ± 10.56	0.009^a^	0.493
BMI, kg/m^2^	23.89 ± 2.66	24.30 ± 2.82	22.31 ± 2.19	22.57 ± 2.35	0.031^a^	0.605
Fat mass index, kg/m^2^	5.23 ± 2.71	5.70 ± 2.97	5.81 ± 1.94	5.76 ± 2.03	0.439	0.047^b^
Fat-free mass index, kg/m^2^	18.95 ± 3.11	18.34 ± 2.51	16.46 ± 1.88	16.89 ± 2.12	0.004^a^	0.082
Total 25(OH)D, nmol/L	47.37 ± 13.29	83.68 ± 32.98	43.10 ± 22.00	49.22 ± 25.40	0.183	0.001^b^
25(OH)D_2_, nmol/L	2.85 ± 1.83	1.91 ± 2.03	2.17 ± 1.96	2.89 ± 1.90	0.313	0.009^b^
25(OH)D_3_, nmol/L	44.49 ± 13.35	81.77 ± 33.66	40.93 ± 22.09	46.33 ± 25.64	0.225	0.001^b^
Vitamin D intake, µg/day	6.73 ± 5.33	–	4.93 ± 2.47	–	0.227	–
PTH, g/dL	41.21 ± 15.98	42.24 ± 20.96	39.28 ± 19.12	43.93 ± 23.65	0.502	0.204
Adjusted calcium, mmol/L	2.29 ± 0.07	2.29 ± 0.09	2.29 ± 0.08	2.31 ± 0.08	0.961	0.427
Creatinine, mmol/L	98.81 ± 11.51	99.21 ± 14.10	89.20 ± 7.10	90.30 ± 9.33	0.001^a^	0.580
Adjusted eGFR, mL/min/1.73 m^3^	94.10 ± 9.30	94.92 ± 15.80	98.10 ± 9.76	97.10 ± 11.89	0.439	0.522
Physical activity, h/day	1.06 ± 1.04	0.70 ± 0.59	0.88 ± 0.89	0.51 ± 0.47	0.466	0.749
VO_2_ max, mL/kg/min	50.46 ± 6.97	50.41 ± 6.96	51.66 ± 6.79	49.33 ± 5.92	0.961	0.176
FEV_1_, L	3.58 ± 1.03	3.47 ± 0.73	3.13 ± 0.81	3.25 ± 0.86	0.125	0.311
FVC, L	4.20 ± 1.06	3.85 ± 0.92	3.40 ± 0.96	3.58 ± 1.03	0.014^a^	0.004^b^
FEV_1_:FVC, %	86.10 ± 14.44	90.41 ± 6.94	93.02 ± 6.10	92.14 ± 5.41	0.050^a^	0.115
Vertical jump height, cm	31.71 ± 8.32	32.15 ± 8.91	27.36 ± 6.49	28.85 ± 6.99	0.068	0.619
Left handgrip strength, kg	36.78 ± 10.04	36.77 ± 11.05	29.71 ± 11.56	32.98 ± 11.30	0.039^a^	0.076
Right handgrip strength, kg	39.29 ± 10.57	39.51 ± 10.76	30.54 ± 11.33	30.07 ± 11.30	0.013^a^	0.091

*BMI* body mass index, *25*(*OH*)*D* 25-hydroxyvitamin D, *PTH* parathyroid hormone, *eGFR* estimated glomerular filtration rate, *VO*_2_ *max* maximal oxygen uptake, *FEV*_1_ forced expiratory volume at 1 s, *FVC* forced vital capacity, *FEV*_1_:*FVC* ratio of forced expiratory volume at 1 s to forced vital capacity

^a^Significant difference between groups at baseline using an independent *t* test, *P* < 0.05

^b^Significant change over time between groups using an ANOVA for normally distributed and transformed data, *P* < 0.05

**Table 2:** Effect of intervention on outcome measures at week 12 expressed as mean ± standard deviation

Treatment group	Vitamin D (*n* = 22)	Placebo (*n* = 20)	ANCOVA test statistics		
Dependent variable	Change from baseline	Change from baseline	*F*	*r* ^2^	*P*
Total 25(OH)D, nmol/L	36.31 ± 32.34	6.11 ± 23.93	15.98	0.396	0.006^a, b^
VO_2_ max, mL/kg/min	−0.64 ± 1.04	−2.00 ± 0.96	1.12	0.535	0.375^c^
FEV_1_, L	−0.12 ± 0.73	0.12 ± 0.55	0.36	0.520	0.665^d^
FVC, L	−0.35 ± 0.73	0.18 ± 0.66	1.59	0.569	0.573^d^
Vertical jump height, cm	0.42 ± 6.59	1.51 ± 6.95	0.09	0.350	0.797^e^
Left handgrip strength, kg	−0.03 ± 5.51	3.30 ± 5.98	2.40	0.665	0.146^e^
Right handgrip strength, kg	0.18 ± 5.64	3.56 ± 6.69	1.43	0.627	0.266^e^

25(*OH*)*D* 25-hydroxyvitamin D, *VO*_2_ *max* maximal oxygen uptake, *FEV*_1_ forced expiratory volume at 1 s, *FVC* forced vital capacity

^a^Significant effect of treatment after adjusting for covariates, *P* < 0.05

^b^Adjusting for total 25(OH)D concentration pre-intervention

^c^Adjusting for VO_2_ max pre-intervention and change in physical activity and FFMI

^d^Adjusting for FEV_1_ or FVC pre-intervention, BMI and change in physical activity

^e^Adjusting for vertical jump height or handgrip strength pre-intervention, change in FFMI, FMI and physical activity

## Discussion

This is not only the largest investigation of vitamin D supplementation on VO_2_ max in athletes to date but also the first randomised controlled trial to successfully optimise vitamin D status using an oral spray solution. In this study, over half of the total cohort presented with vitamin D insufficiency or deficiency at baseline (50 and 22 %, respectively). Such findings corroborate existing literature [3, 5], reiterating that athletes are a population that may be at risk of poor bone health, owing to total 25(OH)D concentrations below 50 nmol/L [63, 64]. Our previous research identified this health concern in elite Gaelic footballers, a finding now corroborated in those competing at the collegiate level [3]. Twelve-week vitamin D_3_ supplementation using a 3000 IU (75 µg) oral spray solution resolved vitamin D deficiency and increased mean total serum 25(OH)D concentrations to over 80 nmol/L. Conversely, those allocated to the PL treatment group remained at potential risk of poor bone health with a mean total 25(OH)D concentration below 50 nmol/L at week twelve [65]. In contrast, Storlie et al. [66] reported no significant change in total 25(OH)D concentrations over 12 weeks in athletes supplemented daily with a 1000 IU (25 µg) vitamin D_3_ oral spray compared to placebo, supporting 3000 IU (75 µg) as an effective wintertime dosage. There is ongoing debate over what constitutes an optimal total 25(OH)D concentration for athletes. Some speculate that higher total 25(OH)D concentrations in excess of 100 nmol/L may be necessary to trigger the purported extra-skeletal benefits of vitamin D in athletes [67, 68], though there is currently a lack of strong evidence for a benefit of maintaining total 25(OH)D concentrations above this threshold [35]. Furthermore, toxicity manifests itself as hypercalcaemia and increased bone resorption at vitamin D supplementation doses exceeding 10,000 IU/day (250 µg) or a total 25(OH)D concentration ≈750 nmol/L [69]. Indeed, there was a significant change in FMI over the 12-week intervention period. FMI increased in the VD group but declined in the PL group. Although in-season, this finding may be explained by differences in the footballers’ individual training and conditioning programmes during the winter months in the absence of scheduled training sessions at the university.

Mechanistic studies support the concept that vitamin D may influence VO_2_ max through direct and indirect actions on iron metabolism [24, 25]. Nevertheless, studies investigating the potential link between total 25(OH)D concentration and VO_2_ max in vivo have yielded equivocal findings to date with many failing to account for important covariates such as PTH concentrations and participation in moderate-vigorous physical activity [13, 15, 70–75]. Moreover, universal criteria for VO_2_ max have not been firmly established and therefore this may contribute to variation in study outcomes. Total 25(OH)D concentration was not associated with VO_2_ max at either time point in the current study, a finding that is supported by Fitzgerald et al. 2014 and research conducted in healthy adults [14, 16]. In contrast, at a latitude of 35°N, Koundourakis and colleagues reported positive bivariate correlations between total 25(OH)D concentration and the VO_2_ max of elite footballers, before and after a tapering period spanning the months of June and July [17]. During this time, there was a small yet significant decrease in athlete’s VO_2_ max despite a concomitant increase in total 25(OH)D concentrations, likely owing to a reduced training load. Such findings indicate that vitamin D does not play a supportive role in determining VO_2_ max in athletes, a concept that is substantiated by findings of the current study. Supplementation did not significantly increase VO_2_ max compared to the PL group despite increasing total 25(OH)D concentration by 77 %, a finding that has also been shown by others [76]. This contrasts with some randomised controlled trials of patients with cardiorespiratory pathology, although findings have not been consistent [77, 78]. Established primary determinants of VO_2_ max include cardiac output and oxygen diffusion capacity [21], and a possible explanation for the disparity between athletes and patients may be that athletes have a smaller ability to improve VO_2_ max, owing to significant cardiovascular adaptations to aerobic training including enhanced cardiac output and capillary density [79]. It is plausible that such adaptations may outweigh any potential benefit of vitamin D supplementation on VO_2_ max when compared to patients with diminished cardiac output and/or oxygen diffusion capacity.

The in vitro mechanisms by which vitamin D, specifically 1,25(OH)_2_D, may impact upon the skeletal muscle function of athletes have been reviewed extensively elsewhere, although less is known about the effects of vitamin D supplementation on skeletal muscle function in vivo [7, 80]. Indeed, the effects of vitamin D supplementation on skeletal muscle function in athletes have been investigated before, albeit without taking into account change in physical activity or body composition, confounders that determine skeletal muscle function and may therefore mask a null effect of treatment [81, 82]. Total 25(OH)D concentration was not associated with any measure of skeletal muscle function in the current study, and supplementation did not significantly increase vertical jump height or handgrip strength when compared to PL, corroborating findings from a recent randomised controlled trial conducted in adolescent swimmers [83]. These results demonstrate that vitamin D_3_ supplementation does not enhance skeletal muscle function in younger adults after taking into account change in FMI, FFMI and moderate-vigorous physical activity, despite increasing total 25(OH)D concentrations to over 80 nmol/L. This contrasts with large studies of elderly patients that have identified a beneficial effect of vitamin D and calcium supplementation on skeletal muscle function and risk of falls [84–86]. Aside from differences in the supplementation regime it is possible to speculate, based on the results of the current study and existing literature, that vitamin D_3_ supplementation only benefits skeletal muscle in those with diminished function, explaining why no ergogenic effect was observed in this cohort of trained footballers. Another consideration is that the time taken for supplementation to increase total 25(OH)D concentrations to over 50 nmol/L, within the 12-week intervention, is not known. Therefore, it is possible that supplementation only increased total 25(OH)D concentrations to above 50 nmol/L late in the intervention, leaving little time for a measurable effect on skeletal muscle function parameters.

Emerging research also posits that total 25(OH)D concentrations may be related to lung function, especially in those with airway disease [87–89]. Potential mechanisms include enhanced innate immunity and downregulation of the T helper 1 cell response, resulting in less airway inflammation and a consequent improvement in overall airway function [90]. Nevertheless, this study did not observe an association between total 25(OH)D concentration and measures of lung function at either time point and, in adjusted analyses, supplementation with vitamin D_3_ did not significantly impact upon FEV_1_ or FVC when compared to PL. In unadjusted analyses, there was a significant decrease in FVC over time in the VD group compared to those allocated to PL yet this was not the case in adjusted analyses indicating that the intervention did not have an adverse effect. These findings suggest that the response to vitamin D_3_ supplementation, in terms of benefits on lung function parameters, may differ between healthy individuals and those with airway disease although larger studies, adjusting for lung function specific confounders are required.

To our knowledge this is the first study to investigate the effects of vitamin D_3_ supplementation on VO_2_ max in athletes that has adjusted for key component covariates. Strengths of the study include a gender balance of athletes, accurate recording of compliance, independently verifying the vitamin D_3_ content of supplements and being adequately powered to detect any potential significant change in VO_2_ max. Due to the heterogeneous athlete population it is not known whether our findings translate to non-Caucasian athletes or those from endurance or strength-based disciplines. The VO_2_ max test used in the current study was not validated. Future studies in this area are encouraged to utilise a validated protocol. The sample size for this study was specific to the primary outcome measure, VO_2_ max. Further research with larger sample sizes may therefore be required in order to categorically rule out a beneficial effect of vitamin D supplementation on skeletal muscle and lung function in athletes. Future studies may also wish to consider stratifying by weight as well as sex to prevent differences between treatment groups at baseline.

In conclusion, this study observed a high prevalence of vitamin D insufficiency and deficiency in collegiate Gaelic footballers during wintertime and such individuals should consider vitamin D supplementation to avoid being at risk of poor bone health. Twelve-week daily supplementation with a 3000 IU (75 µg) vitamin D_3_ oral spray solution is an appropriate method, dose and duration to resolve deficiency and increase total 25(OH)D concentrations to over 80 nmol/L. However, vitamin D supplementation, at the dose provided here for 12 weeks, did not have any beneficial effect on VO_2_ max, skeletal muscle or lung function in this cohort of Gaelic footballers.
